# Navigating the Data Gaps of Ageing Among Women Living With HIV

**DOI:** 10.1002/jia2.70094

**Published:** 2026-03-05

**Authors:** Caroline A. Sabin, Nomathemba Chandiwana, Anchalee Avihingsanon, Nicoletta Policek

**Affiliations:** ^1^ Institute for Global Health UCL London UK; ^2^ Desmond Tutu HIV Centre University of Cape Town Cape Town South Africa; ^3^ HIVNAT, Thai Red Cross AIDS and Infectious Disease Research Centre and Center of Excellence in Tuberculosis, Faculty of Medicine Chulalongkorn University Bangkok Thailand; ^4^ European AIDS Treatment Group Brussels Belgium

1

In its 2025 Global AIDS Update [1], UNAIDS estimates that over half (53%) of the 40.8 million people living with HIV globally were women or girls, who also accounted for 45% of new HIV acquisitions. This sex‐based disparity is pronounced in Africa, where women and girls accounted for 63% of all new acquisitions. Where women are diagnosed and receive optimal antiretroviral therapy (ART), life expectancy has improved. As women live longer with HIV, prolonged ART exposure and chronic immune activation increasingly intersect with ageing, bringing an increased risk of age‐associated non‐communicable conditions (AANCCs), including cardiovascular, metabolic, bone and renal disease.

Women may experience a unique range of AANCCs compared to men. In the Dutch ATHENA cohort, women developed multi‐morbidity at younger ages than men, with higher mortality rates in women following each morbidity event [2]. In the United States, a combined analysis of the Women's Interagency HIV Study and the Multicenter AIDS Cohort Study demonstrated increasing multi‐morbidity with age in both sexes, regardless of HIV status. However, among those with HIV, women consistently exhibited a higher comorbidity burden at all ages. Importantly, women had higher rates of bone and lung disease (primarily asthma) and diabetes, but lower rates of hypertension, psychiatric illness, dyslipidaemia, liver disease and non‐AIDS cancers when compared to men [3]. Differences in co‐morbidity profiles are projected to continue until at least 2030 [4], although profiles vary by ethnicity and injection drug use, emphasizing the contribution of social and structural inequalities to AANCC development [3]. Women in the D:A:D/RESPOND cohort collaboration [5] experienced high rates of human papillomavirus‐related, breast and lung cancers, as well as non‐Hodgkin's lymphoma, with geographical variations in some cancers, particularly cervical cancer, raising concern about differential access to vaccination and other preventive interventions.

Over the past decade, while we have learned much about the ageing process in men living with HIV, the same cannot be said for women. A systematic review of published articles from 2010 to 2020 [6] confirmed a high burden of age‐related morbidity among women with HIV, including acute cardiovascular disease, reduced bone mineral density, cognitive impairment and renal disease. However, the review highlighted substantial gaps in our understanding of why these differences exist, noting poor female representation in studies with few sex‐stratified analyses, a lack of geographical diversity, and inadequate adjustment for key socio‐demographic and lifestyle confounders, limiting interpretability.

Differences in AANCC among women may partly reflect sex‐hormone‐related factors, which change at key times during a woman's life, including pregnancy, lactation and menopause. Even in the absence of HIV, bone mineral density declines during these times with additional impacts of some ART and contraceptive drugs [7]. Menopause represents a critical cardiometabolic transition, during which increases in central adiposity, insulin resistance, adverse lipid profiles and endothelial dysfunction collectively heighten the risks of many AANCCs [8, 9]. Whether menopause occurs earlier among women living with HIV remains unclear, as studies reporting earlier onset often fail to account for key determinants such as social factors and ethnicity [10]. Regardless of age at onset, menopause has a huge impact on the physical and mental health of women and has been associated with poorer adherence to ART and reduced quality‐of‐life. Drops in quality‐of‐life occur as women move from the pre‐ to peri‐ and post‐menopause stages, with symptom severity a strong driver [11]. Mental health conditions may often go under‐recognized or undiagnosed, and women face additional barriers to care due to stigma associated with both HIV and mental health [12]. In the POPPY study in the United Kingdom and Ireland, a striking difference was seen in the proportion of men and women with moderate or severe depressive symptoms (36% vs. 50%, respectively) as well as lower diagnoses and uptake of mental healthcare in women [13].

Changes in weight and body shape, long associated with ageing and menopause, have gained renewed attention in the context of ART. In the ADVANCE trial, women gained more weight than men across all ART regimens, with weight gain continuing over 5 years of follow‐up. Although the greatest increases were observed among women receiving dolutegravir combined with tenofovir alafenamide, weight gain occurred across regimens, indicating that antiretroviral drug choice alone does not fully explain the observed sex differences [14]. Sex‐related differences in body composition and fat distribution may additionally contribute to higher rates of metabolic disorders, particularly diabetes, sarcopenia and frailty, in women [15]. Together, these findings highlight a complex interplay between biological ageing, hormonal transition, mental health and ART exposure in shaping long‐term metabolic risk.

Older women living with HIV also demonstrate distinct neurocognitive profiles compared to men with greater vulnerability in processing speed, attention and executive functioning [16], even after controlling for socio‐demographic and clinical factors. These disparities may reflect the higher prevalence of depression, early life psychosocial factors and trauma exposure, sleep disturbance, and reduced cognitive reserve among women living with HIV [17]. Clinicians, however, may misattribute cognitive complaints to menopausal changes, delaying appropriate evaluation for these complaints.

In Africa, women living with HIV may experience a potentially accelerated ageing trajectory driven by the convergence of chronic HIV‐related inflammation, ART‐associated metabolic shifts, and high and rising background prevalence of obesity. Clinical patterns suggest earlier onset of cardiometabolic, vascular and cognitive conditions among mid‐life women; however, the underlying biology remains poorly characterized. Rapid population ageing in Africa is occurring in the absence of longitudinal ageing cohorts, and African women, particularly those with HIV, are largely absent from geroscience research, limiting the ability to distinguish HIV‐related ageing effects from background population risk or social adversity [18]. In Asia, ageing among women with HIV is shaped by rapid demographic transition, a high burden of cardiometabolic disease, lower and more rapidly declining bone mineral density, limited access to menopause‐related care and persistent gender norms that prioritize caregiving over self‐care [19, 20]. In Thailand, in particular, older women living with HIV often balance family caregiving responsibilities with chronic illness, stigma and financial insecurity, yet remain under‐represented in research, policy and health service design.

Globally, persistent evidence gaps are contributing to widening sex‐based inequalities in the support provided to those ageing with HIV. Despite their central caregiving roles within families and communities, older women remain under‐represented in research cohorts, inadequately captured in sex‐ and age‐stratified analyses, and poorly served by health systems that are rarely designed to address the intersecting effects of ageing, menopause and long‐term HIV care. These gaps reflect structural features of research, policy and service delivery, including eligibility criteria, analytic priorities and limited integration of women‐centred ageing care, rather than biological inevitability. Addressing these inequities will require deliberate changes in research practice, clinical service design and policy implementation, as outlined in Table 1.

**TABLE 1:** Priority recommendations to address ageing‐related non‐communicable diseases among women living with HIV.

Research partnership	Engage women living with HIV as equal partners in identifying evidence gaps, setting research priorities and interpreting findings.
Sex‐specific pathways	Design analyses to test sex‐specific pathways to comorbidity development, presentation and treatment, rather than treating sex as a confounder.
Women‐specific data	Routinely collect women‐specific data, including menopausal status, reproductive history, and breast and cervical cancer screening and treatment.
Representation	Ensure adequate representation of women with sufficient sample size for meaningful sex‐stratified analyses; provide practical support to enable participation.
Risk measurement	Improve cardiovascular risk assessment in women by moving beyond body mass index‐based approaches to include measures of adiposity distribution, insulin resistance, vascular dysfunction and early cardiometabolic risk.
Evidence gaps	Conduct studies to: address stigma, mental health and the experiences of gender‐based violence in women, and to understand how these influence healthcare access, adherence and comorbidity outcomes; and examine and evaluate the contribution of caregiving burden to psychological stress, socio‐economic vulnerability, healthcare utilization, treatment adherence and participation in research.

## Author Contributions

CAS drafted the initial version of the manuscript, with the input of all other authors. All authors have reviewed and approved the final submitted version.

## Conflicts of Interest

CAS has received funding for the preparation and delivery of educational materials and for participation in advisory panels from Gilead Science, ViiV Healthcare and MSD. NC has received funding for the preparation and delivery of educational materials and travel support from Novo Nordisk.

## Data Availability

Data sharing not applicable to this article as no datasets were generated or analysed during the current study.
